# Count-based method for specific binding ratio calculation in [I-123]FP-CIT SPECT analysis

**DOI:** 10.1007/s12149-018-1297-1

**Published:** 2018-10-01

**Authors:** Mahmudur G. M. Rahman, Muhammad M. Islam, Tetsuya Tsujikawa, Yasushi Kiyono, Hidehiko Okazawa

**Affiliations:** 1 Biomedical Imaging Research Center, University of Fukui, 23-3, Matsuoka-Shimaizuki, Eiheiji, Fukui 910-1193 Japan; 2 Department of Biomedical Engineering, Khulna University of Engineering and Technology, Khulna, Bangladesh

**Keywords:** [I-123]ioflupane (FP-CIT) SPECT, DAT, SBR, ACSC, CTAC

## Abstract

**Objective:**

To calculate the specific binding ratio (SBR) appropriately in dopamine transporter (DAT) imaging, a method for extracting the striatal volume of interest (VOI) was developed.

**Methods:**

This study included 200 patients (72 ± 10 years) who were suspected of parkinsonian syndromes (PS) or dementia with Lewy body (DLB). The patients were divided into three groups of PS with dopaminergic degeneration, DLB and non-PS after [^123^I]ioflupane (FP-CIT) SPECT and clinical follow-up. The image data were reconstructed with CT attenuation correction and scatter correction, and with only CT attenuation correction (CTAC). The new method extracted striatal VOI according to the high-level counts and the average striatum volume, and calculated SBR using the reference occipital counts. The SBR values for each patient were obtained using the Tossici-Bolt method (SBR_Bolt_) and our method. Reproducibility of SBR calculation using our method was compared by two operators.

**Results:**

The mean SBR values for the PS and DLB groups were significantly different from that of the non-PS group with both methods. The coefficients of variation of the SBR were significantly smaller with the proposed method compared with those of SBR_Bolt_ (*p* < 0.001), except for the CTAC images. There were no differences in SBR between the two operators using our method. The diagnostic accuracies with our method for the PS and DLB groups were 98.4 and 96.0%, respectively.

**Conclusion:**

Our new method for SBR calculation in the FP-CIT SPECT showed less coefficients of variation with high reproducibility, which would be useful for clinical diagnosis and in assessing the severity of diseases in follow-up studies.

## Introduction

Dopamine transporter (DAT) imaging using [^123^I]ioflupane (FP-CIT) SPECT is widely used for clinical assessment of the nigrostriatal function in patients with parkinsonian syndromes (PS) [1–4]. To evaluate presynaptic functional reduction of the striata, the specific binding ratio (SBR), a semi-quantitative value of FP-CIT accumulation, is calculated using various methods. Many quantitative methods have been proposed to assist with visual image interpretation in cases of movement disorder with parkinsonism because visual evaluation relies on the observer’s ability and may induce inter- and intra-observer inconsistency [5]. Semi-quantitative evaluation would be useful for assessing disease progression, evaluating disease severity, and observing the clinical outcome after treatment [6–8]. Since a method with manual region of interest (ROI) selection revealed intra- and inter-observer variability [5], a three-dimensional (3D) automatic semi-quantification method was developed and used for data analysis in multicenter studies [9]. A 3D-ROI semi-quantification software program named ‘BasGan’ is now very commonly used in Europe [9, 10]; the shape and location of the basal ganglia are automatically determined based on the Talairach atlas. Another sophisticated method, the Tossici-Bolt (TB) method, is also widely used for SBR calculation [11]. In this method, a large size ROI including the striatum is selected to avoid the partial volume effect (PVE), and the intra- and inter-operator variability were reported to be small (3–4%). However, the TB method provides 2- to 3-fold greater SBR values compared with other methods, and thus a large variability with substantial overlapping in the semi-quantitative values of the striatal tracer accumulation between patient groups and healthy subjects.

In this study, a new method for extraction of the striatal volume of interest (VOI) was developed to calculate the SBR appropriately. The proposed method has a semi-automatic procedure with an easy and operator-friendly process for accurate measurement of SBR. The results of SBR with our method were compared with those from the TB method, the most common method for semi-quantitative SBR evaluation in Japan, and also evaluated for inert-operator variance.

## Materials and methods

### Subjects

Two hundred patients (91 males and 109 females, 72 ± 10 years) with suspected of PS or dementia with Lewy body (DLB) were included in this study. They were referred to the Departments of Neurology or Psychiatry in our University Hospital and underwent [^123^I]FP-CIT SPECT for differential diagnosis of movement disorder or dementia. The patients were divided into three groups, PS with dopaminergic degeneration (*n* = 101), DLB (*n* = 11) and non-PS (*n* = 88), after the [^123^]FP-CIT SPECT, perfusion SPECT and clinical follow-up for more than 12 months. The PS group consisted of Parkinson’s disease, multiple system atrophy, progressive supranuclear palsy and corticobasal degeneration/syndrome This study was designed retrospectively to improve the accuracy of SBR evaluation using FP-CIT SPECT data. The protocol of this study was approved by the Ethics Committee of our University, Faculty of Medical Sciences (#20170225).

### SPECT imaging

Patients underwent SPECT scans after intravenous slow injection of about 170 MBq [^123^I]FP-CIT in the morning (167 MBq at noon). The scan was started about 3–4 h after the injection using a SPECT/CT scanner (Symbia T2, Siemens, Erlangen, Germany) with a dual-head gamma camera and low- and medium-energy general-purpose (LMEGP) collimators. The settings of SPECT/CT scans were the same as in the previous study [12]. In brief, the gamma camera was calibrated for a 159-keV photo-peak and ± 10% energy window. Two sub-windows for triple-energy window scatter correction (SC) were set as a 7% energy window on the both upper and lower sides of the photo-peak window. SPECT data acquisition was performed in 45 frames with four cycles of 210 s/cycle scan for a 180° angular range in steps of 4°. The two camera heads were positioned as close as possible to the patient’s head. A low-dose CT scan for attenuation correction was performed after SPECT data acquisition.

SPECT data were reconstructed by an iterative algorithm using 3D-ordered subset expectation maximization (OSEM) with eight iterations and ten subsets. Two image sets were reconstructed from the image data, i.e., with CT attenuation correction and SC (ACSC), and with only CT attenuation correction and no SC (CTAC). After 1.5× expansion, the pixel size of each slice was 2.20 mm in a 128 × 128 matrix, and a 6.0-mm Gaussian filter was applied.

### New calculation method for SBR

**Fig. 1:**
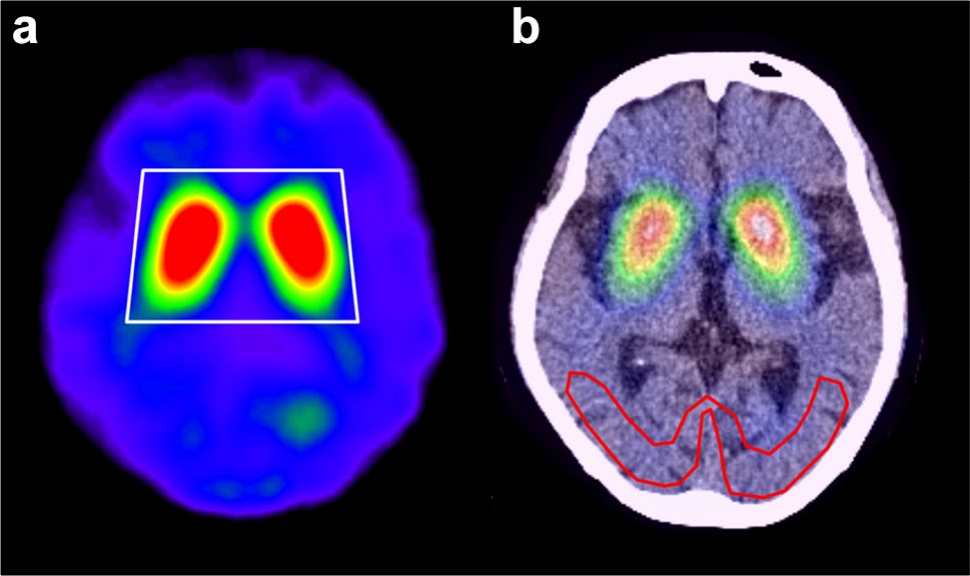
Striatal trapezoid VOI (VOI_t_) location at the slice level of the striatal centre on DaT-SPECT (**a**). The VOI_t_ includes the whole striatum for both sides. To obtain the background activity, the background VOI (VOI_bg_) was drawn on the occipital cortex using several image slices avoiding the sinus region and the cerebrospinal space (**b**)

**Fig. 2:**
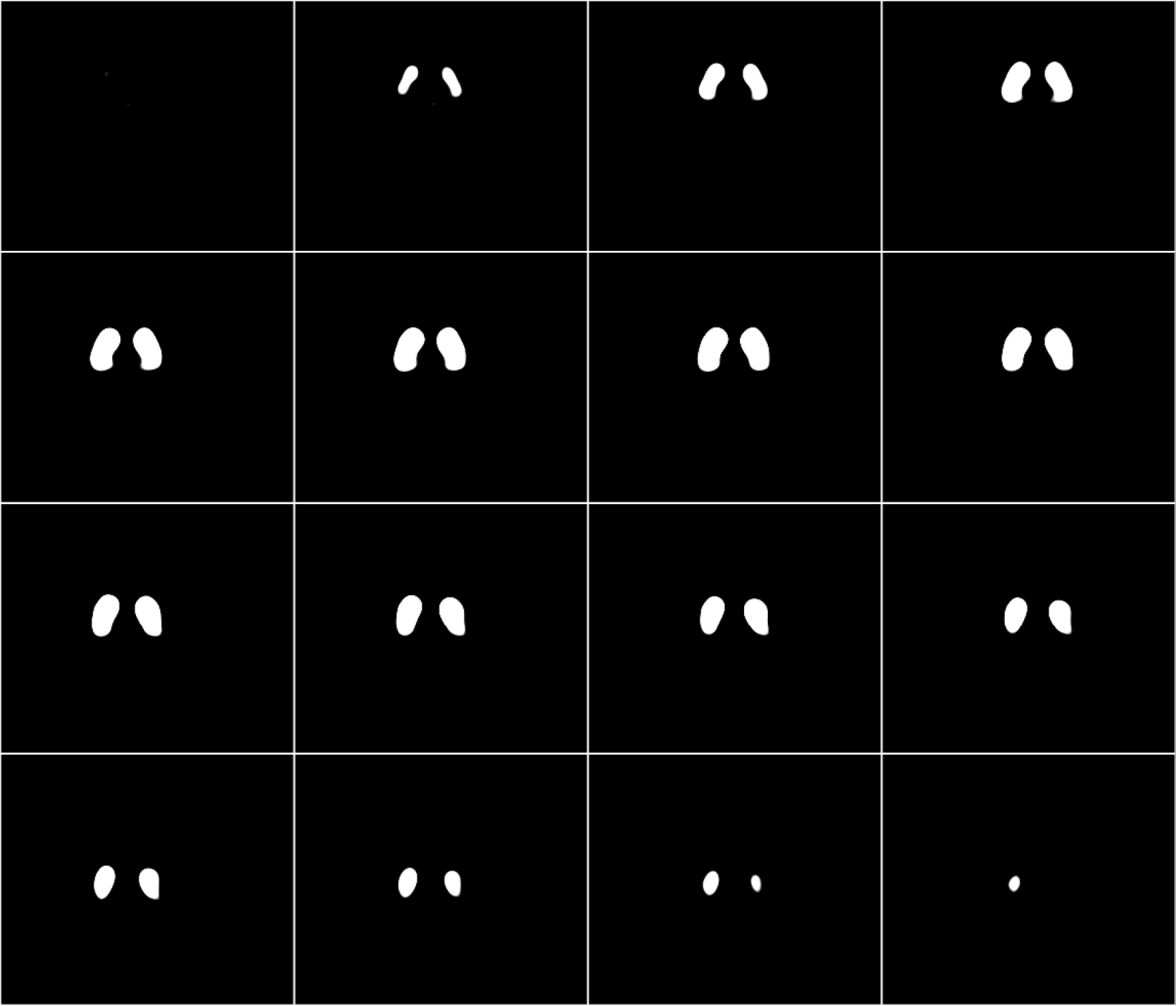
Representative striatal VOI mask slices determined based on the intense voxel activity of the DAT image. A cluster of 1052 high-intensity voxels (11.2 mL) was extracted consecutively from the maximal count of each side in the VOI_t_ and saved as the striatal VOI mask (VOI_st_) to obtain the bilateral striatal counts

SBR can be defined as the ratio of striatal count concentration due to specific binding in the striatum to the non-specific binding in the background. The proposed method is based on the calculation of the specific radioactivity concentration from the most-intense voxel in the striatal region. First, the range of slices that included the bilateral striata was determined for each patient by visual observation. A large trapezoid VOI (VOI_t_) with adequate thickness completely covering the bilateral striata was determined first by drawing manually on the centre slice level (Fig. 1a), and then applied to all slices covering the whole striatum. This VOI_t_ was set only to indicate the striatal position, but not to be used for SBR calculation. The standard volume of the striatum is reported as 11.2 mL [11, 13, 14], which includes 1052 voxels for each side in the SPECT image with a voxel size of 2.2 mm × 2.2 mm × 2.2 mm. We extracted consecutive 1052 high-intensity voxels from the maximal count of each side in the VOI_t_ and saved this as the striatal VOI mask (VOI_st_) to obtain the bilateral striatal counts separately (Fig. 2). In this step, the right-side peak voxel was determined first from the right half area in the VOI_t_, followed by extraction of the left half peak voxel. VOIs for background activity (VOI_bg_) were manually selected in the occipital region using a couple of slices (Fig. 1b) and the VOI_bg_ mean count was obtained for the non-specific background count. The SBR was calculated using the count concentration and the following equation:

$${\text{SBR}}=\frac{{\left[( {{\text{Total}}\;{\text{VO}}{{\text{I}}_{{\text{st}}}}\;{\text{count}})/{\text{ }}11.2{\text{ }}-({\text{VO}}{{\text{I}}_{{\text{bg}}}}\;{\text{mean count}}}) \right]{\text{ }}\left( {{\text{/mL}}} \right)}}{{{\text{VO}}{{\text{I}}_{{\text{bg}}}}\;{\text{meancount}}\left( {{\text{/mL}}} \right)}}.$$

### Statistical analysis

The SBR values obtained by the new method were compared with those from the TB method (SBR_Bolt_) [11] obtained using a specific software program (DaT View, AZE Inc., Tokyo, Japan). Two operators separately analysed the DAT image data using our proposed method. In the PS group, the SBR values of the dominant side were used for analysis. In the DLB and non-PS groups, the average values of the bilateral SBR were compared. Statistical analysis was performed using SPSS Version 18.0, and *p* < 0.05 was considered significant. Differences between groups were evaluated by the analysis of variance (ANOVA), and repeated measures ANOVA was applied for comparison of reconstruction methods. Both statistical analyses were followed by post hoc tests after detection of group differences. To evaluate difference in diagnostic accuracy using SBR values, receiver operating characteristic (ROC) analysis was used and area under the curve (AUC) was statistically compared using *χ*^2^-test.

## Results

**Fig. 3:**
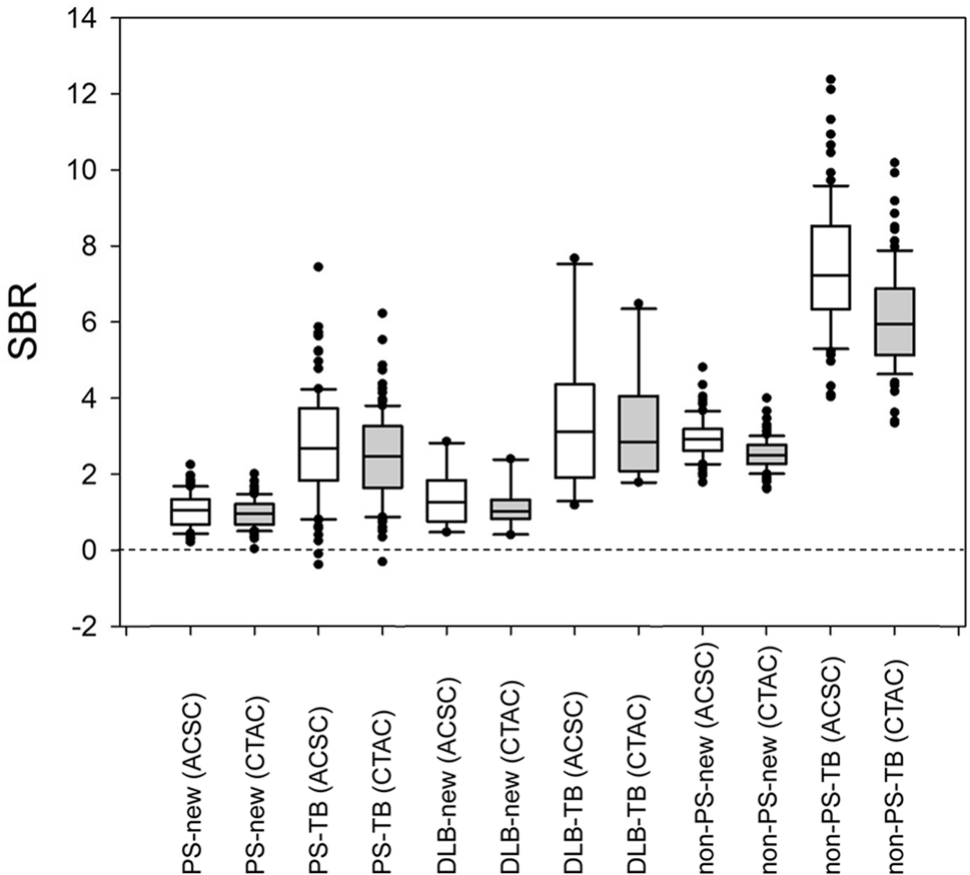
Box-and-whisker plots for SBR values of the three groups (PS, DLB and non-PS) obtained by the two methods (our new method and TB method) and two reconstructions (ACSC and CTAC). PS and DLB groups showed significantly lower SBR than non-PS with both methods although the SBR values from our method were significantly lower than SBR_Bolt_ from the TB method

**Fig. 4:**
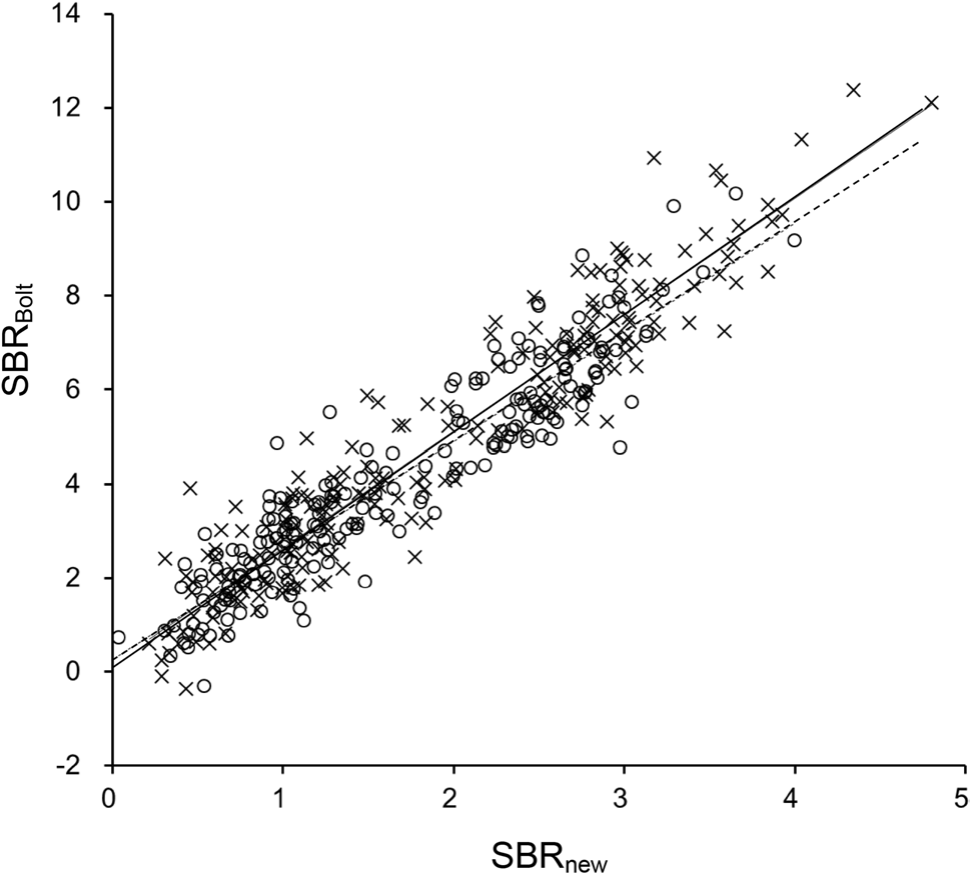
Scatter plots of SBR values from the TB method (SBR_Bolt_) and our new method (SBR_new_). Solid (*y* = 2.5*x* + 0.16, *r* = 0.95) and dashed (*y* = 2.3*x* + 0.27, *r* = 0.93) lines show good linear correlations between the two SBRs for ACSC (x) and CTAC (unfilled circles), respectively

There were no differences in mean age (72 ± 10 years) among the three groups of PS, DLB and non-PS. Table 1 shows the SBR, SBR_Bolt_ values and coefficient of variation (CV) for each group and the different methods for SBR calculation and image reconstruction. Patient groups showed significant differences in SBR when compared with the non-PS group (*p* < 0.0001) for both our new method and the conventional TB method. The ACSC reconstruction showed significantly greater SBR values than the CTAC reconstruction with both methods. The TB method showed 2- to 3-fold greater SBR values (SBR_Bolt_ vs. SBR) as well as greater variance compared with the new method (Fig. 3; Table 1). The CVs of the SBR from the new method were significantly smaller compared with those of SBR_Bolt_ (*p* < 0.001, *F* test), except for the CTAC reconstruction images in the DLB group. The repeated measures ANOVA between two operators showed no significant difference within the same reconstruction method according to our new method (*p* > 0.79). Figure 4 shows scatter plots comparing SBR values from the two methods. Both ACSC and CTAC images showed good linear correlations (*r* = 0.95 and 0.93, respectively) between them.

**Table 1:** SBR values from two methods and two reconstruction images

Groups	New-ACSC	New-CTAC	Bolt-ACSC	Bolt-CTAC
PS (*n* = 101)	1.05 ± 0.45^†,^*	0.97 ± 0.37^†,^*	2.78 ± 1.41^†,^*	2.49 ± 1.17^†,^*
CV	0.42	0.39	0.51	0.47
DLB (*n* = 11)	1.42 ± 0.80^†,^**	1.20 ± 0.65^†,^**	3.63 ± 2.05^†,^*	3.39 ± 1.55^†,^*
CV	0.56	0.54	0.57	0.46
Non-PS (*n* = 88)	2.97 ± 0.54*	2.54 ± 0.42*	7.48 ± 1.72*	6.12 ± 1.36*
CV	0.18	0.16	0.23	0.22

Values are means ± SD

New- and Bolt- are SBR calculation methods; the proposed new method and the Tossici-Bolt method, ACSC and CTAC are reconstruction methods with or without scatter correction

*CV* coefficient of variation

^†^*p* < 0.0001 in comparison with non-PS group

^*^p < 0.0001, ^**^p < 0.01 comparing ACSC and CTAC

**Fig. 5:**
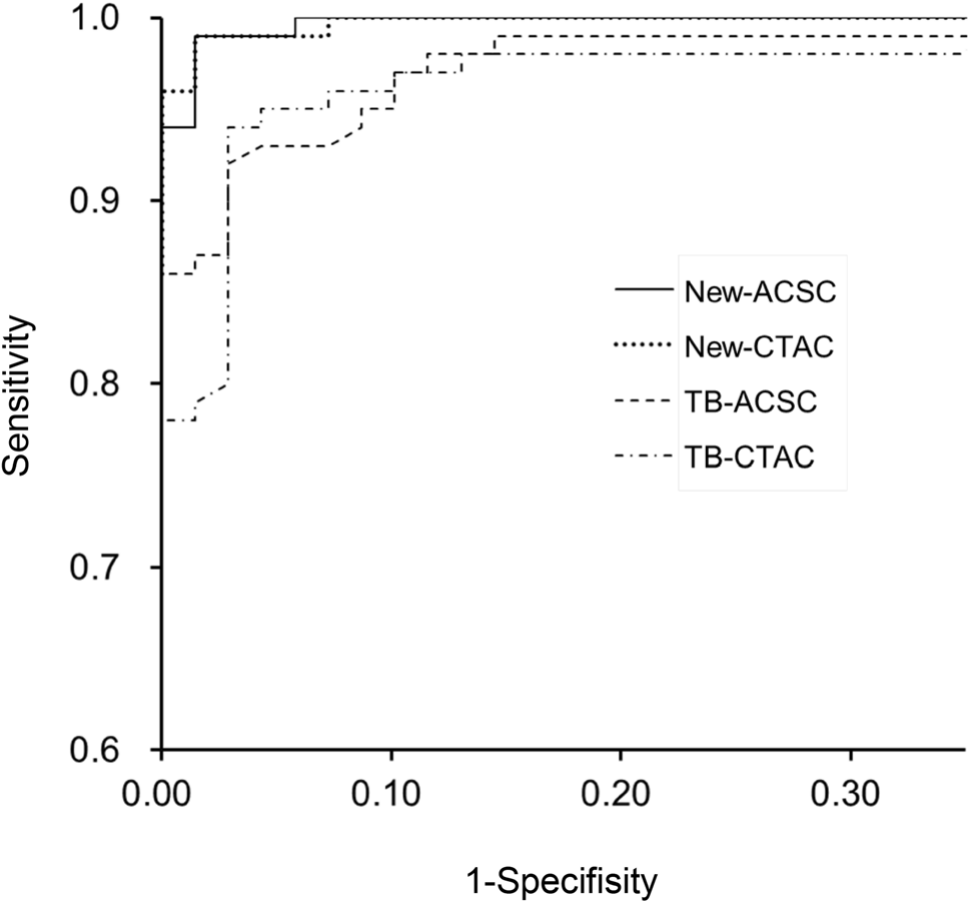
ROC analysis for evaluation of diagnostic accuracy was performed and ROC curves showed greater AUCs of our new method (> 0.998) compared with those of the TB method (ACSC 0.987, CTAC 0.983)

ROC analysis for evaluation of diagnostic accuracy was performed and it showed greater AUC of the ROC curve in our new method (> 0.998) than that from the TB method (< 0.987) for both ACSC and CTAC images (*p* < 0.05, Fig. 5). Taking cut-off values of SBR of 2.0 for ACSC and 1.8 for CTAC, which were determined by ROC analysis, the new method could distinguish the patient groups with a diagnostic accuracy of 98.4% for PS and 96.0% for DLB. CTAC-SBR_Bolt_ with a cut-off value of 4.50, determined from a previous report [11], showed a diagnostic accuracy of 94.2% for our PS group and 91.8% for DLB.

## Discussion

DAT imaging using [^123^I]FP-CIT SPECT is very useful in the clinical setting to evaluate presynaptic striatal function in patients with parkinsonian syndrome and DLB [3, 15]. Quantitative analysis has been expected as supporting information for clinical diagnosis, especially in borderline cases whose reduction of tracer accumulation is not definitive on visual observation. The SBR is widely used as a semi-quantitative index for assessment of striatal dopaminergic deficit (SDD) [16, 17]. However, simplified methods such as the TB method sometimes provide unstable values although they may be able to avoid errors induced by the PVE [11, 18]. An automatic procedure may reduce observer variability caused by anatomical variations induced by atrophy or ventricular enlargement [5]. In this study, we proposed a new simplified method to easily obtain relatively stable SBR with good reproducibility. In the count-based method, the striatal VOI mask was well estimated by selecting the consecutive 1052 most-intense voxels based on the standard striatum size. An appropriate VOI mask selection method is a key to good reproducibility of SBR quantification. The new semi-automatic quantification method takes less time than the TB method and is significantly shorter than the manual method.

The SBR, the specific to non-specific radioactivity concentration, was calculated from the counts of extracted voxels (VOI_st_) and those of the reference region (VOI_bg_). In this study, the raw data were reconstructed into ACSC and CTAC image data, and compared among the three groups of PS, DLB and non-PS. The variability of SBR was smaller in the CTAC than the ACSC reconstruction in both methods because scatter correction includes greater variance in the regional counts. Although our method can be applied to non-AC reconstruction images, they were not included in the present study because of the greater variance in SBRs compared with AC images.

The large difference in SBRs between the TB method and our new method was caused simply by the difference in the calculation procedure. In the TB method, the SBR_Bolt_ is calculated as follows:

$${\text{SB}}{{\text{R}}_{{\text{Bolt}}}}={\text{ }}{{\left( {{Q_{{\text{voi}}}}/{C_{\text{r}}} - {\text{ }}{V_{{\text{voi}}}}} \right)} \mathord{\left/ {\vphantom {{\left( {{Q_{{\text{voi}}}}/{C_{\text{r}}} - {\text{ }}{V_{{\text{voi}}}}} \right)} {{V_{\text{s}}}}}} \right. \kern-0pt} {{V_{\text{s}}}}}={\text{ }}{{\left( {{Q_{{\text{voi}}}} - {\text{ }}{C_{\text{r}}}{V_{{\text{voi}}}}} \right)} \mathord{\left/ {\vphantom {{\left( {{Q_{{\text{voi}}}} - {\text{ }}{C_{\text{r}}}{V_{{\text{voi}}}}} \right)} {{C_{\text{r}}}{V_{\text{s}}}}}} \right. \kern-0pt} {{C_{\text{r}}}{V_{\text{s}}}}},$$

where *Q*_voi_ is the total count of striatal VOI, *C*_r_ (count/mL) is the count concentration of the reference region, and *V*_voi_ and *V*_s_ are the volume of striatal VOI in the TB method and the average striatal volume (11.2 mL) [11, 19]. Since *Q*_voi_ is equal to *C*_voi_ (count concentration of striatal VOI) × *V*_voi_, the equation can be modified as follows:

$${\text{SB}}{{\text{R}}_{{\text{Bolt}}}}={\text{ }}{{\left( {{C_{{\text{voi}}}}{V_{{\text{voi}}}}} \right)} \mathord{\left/ {\vphantom {{\left( {{C_{{\text{voi}}}}{V_{{\text{voi}}}}} \right)} {\left( {{C_{\text{r}}}{V_{\text{s}}}} \right)}}} \right. \kern-0pt} {\left( {{C_{\text{r}}}{V_{\text{s}}}} \right)}} - {{{V_{{\text{voi}}}}} \mathord{\left/ {\vphantom {{{V_{{\text{voi}}}}} {{V_{\text{s}}}}}} \right. \kern-0pt} {{V_{\text{s}}}}}={\text{ }}\left( {{V_{{\text{voi}}}}/{V_{\text{s}}}} \right)\left[ {{C_{{\text{voi}}}}/{C_{\text{r}}} - {\text{ }}1} \right]{\text{ }}=R \cdot \left[ {{C_{{\text{voi}}}}/{C_{\text{r}}} - {\text{ }}1} \right],$$

where *R* is a constant ratio of *V*_voi_/*V*_s_. Because the size of *V*_voi_ is fixed in the TB method (44 mm thickness), SBR_Bolt_ is usually greater than the SBR values from the manual method or other automatic method (Fig. 4). The TB method assumes that the striatal radioactivity of [^123^I]FP-CIT exists only inside the striata and the background outside them is almost homogeneous. The theory is perfectly correct under this assumption and SBR_Bolt_ should be identical to other SBR values; however, in the real SPECT images, various factors affect the VOI counts. Since C_voi_ is an average of heterogeneous regional counts affected by counts outside of the striatum including spillover, and PVE caused by inclusion of cerebrospinal fluid (CSF) space or other structures [20], [*C*_voi_/*C*_r_ − 1] in the TB method is not necessarily identical to the SBR values from other methods. This is why the SBR values from the TB method were not exactly *R*-fold greater than those of our method; however, Fig. 4 clearly shows linear correlation between them. Furthermore, the large VOI in the TB method induces some fluctuation of *C*_voi_ depending on its location set by different operators, which is one of the reasons of unstable SBR_Bolt_ values compared with other semi-automatic method.

**Fig. 6:**
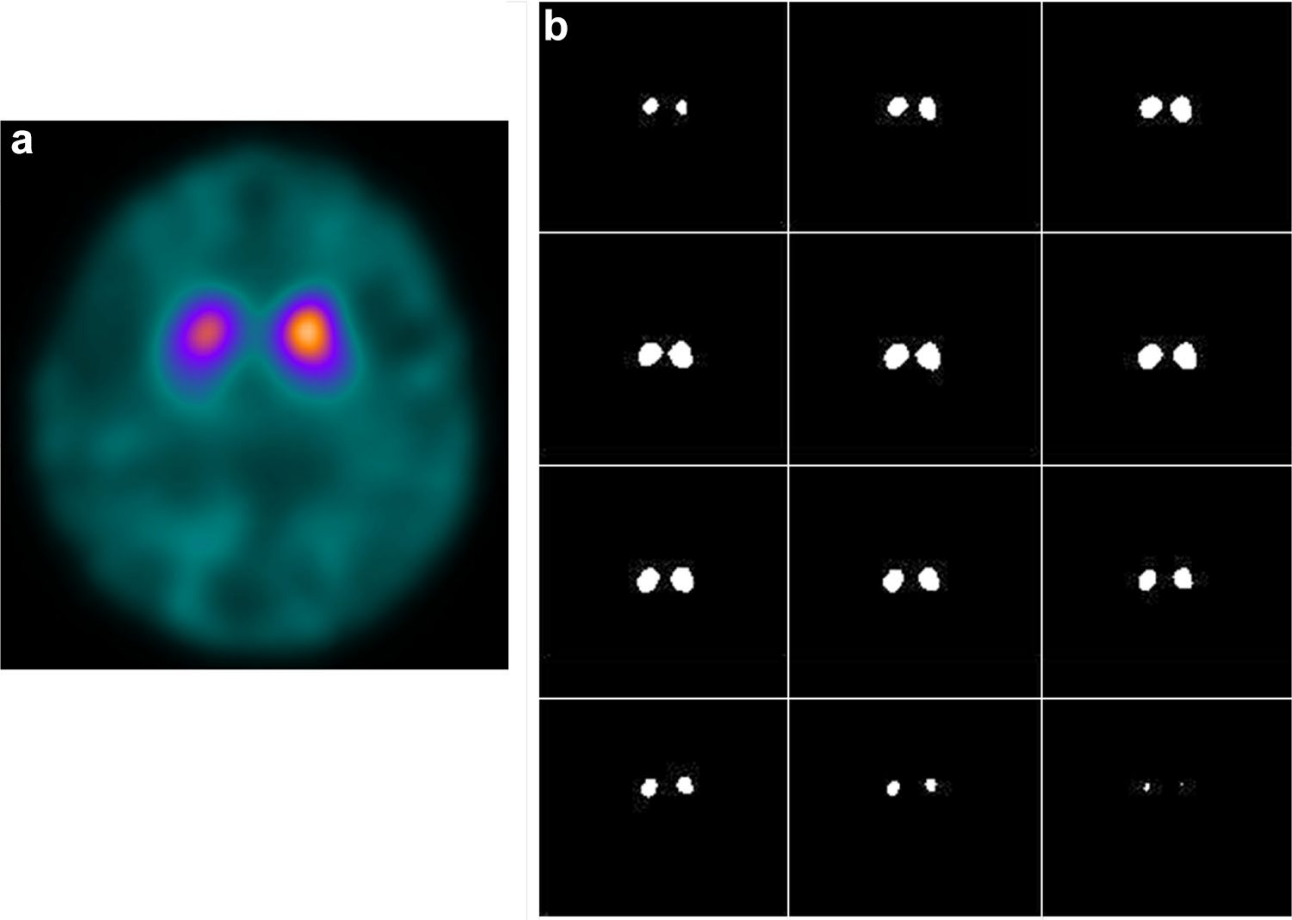
An example of striatal VOI (VOI_st_) in a patient showing right dominant decrease with ‘dot’ pattern (**a**) shows somewhat round-shaped mask due to decrease in putaminal uptake (**b**). Each VOI_st_ consists of the same 1052 voxels

In our proposed method, the striatal mask determined in the VOI_t_ consisted of the 1052 most-intense voxels for each side. Even in cases of reduced striatal accumulation like “dot pattern” and/or lateralized uptake, our method provides the same volume of striatal VOI at an appropriate location (Fig. 6). The mask may include outside structure of the striatum especially in reduced uptake cases; however, a cluster of the consecutive top 1052 voxels was included in the mask and there were no cases whose mask was located apart from the striatum despite very low striatal uptake. Furthermore, low accumulation similar to background does not affect SBR calculation as seen in the equation, because the low-intensity voxel values are almost identical to those of background. The method seems to be very practical and reasonable to place a VOI on the striatum. Recently, a new correction method for SBR_Bolt_ calculation was introduced to avoid PVE from very low counts of the CSF space [21]. The method would reduce the influence of brain atrophy and infarction; however, it cannot avoid the effects of spillover as well as different size of individual *V*_voi_ which induces variation of the constant *R*. Reduction of *V*_voi_ provides smaller *R* than the original one, inducing unstable enhancement factor in the TB method (see the equation above).

The variation in selection of reference regions may be another possible source of variability in SBR results. Researchers have defined various brain regions as the reference, such as the occipital lobes [22–27], the frontal lobes [28–30], and the cerebellum [31, 32]. The occipital lobe is relatively less sensitive to PVE and thereby PVE correction is not required [33]. In the proposed method, variability may be induced only by drawing reference region, although no significant difference was found between two different operators. Recent studies revealed stability in SBR calculation when selecting the whole cerebral VOI except for the striatum as the reference region [34]. In the present study, the difference was negligible between the different reference VOI (data not shown).

## Conclusion

We proposed a new count-based method for SBR calculation in FP-CIT SPECT. This method can extract the striatal count in a simple way, with semi-automatic operation, and provided stable and reproducible results with less variation compared with previous simplified methods. The method will be very useful for calculating SBR in the clinical setting, especially in patient follow-up studies, as well as in multicentre studies.
